# Cerebral Hemodynamics in Patients with Hemolytic Uremic Syndrome Assessed by Susceptibility Weighted Imaging and Four-Dimensional Non-Contrast MR Angiography

**DOI:** 10.1371/journal.pone.0164863

**Published:** 2016-11-01

**Authors:** Ulrike Löbel, Nils Daniel Forkert, Peter Schmitt, Torsten Dohrmann, Maria Schroeder, Tim Magnus, Stefan Kluge, Christina Weiler-Normann, Xiaoming Bi, Jens Fiehler, Jan Sedlacik

**Affiliations:** 1 Department of Diagnostic and Interventional Neuroradiology, University Medical Center Hamburg-Eppendorf, Hamburg, Germany; 2 Department of Radiology and Hotchkiss Brain Institute, University of Calgary, Calgary, Alberta, Canada; 3 Siemens Health Care, Erlangen, Germany; 4 Department of Intensive Care, University Medical Center Hamburg-Eppendorf, Hamburg, Germany; 5 Department of Neurology, University Medical Center Hamburg-Eppendorf, Hamburg, Germany; 6 Department of Internal Medicine, University Medical Center Hamburg-Eppendorf, Hamburg, Germany; 7 Martin Zeitz Center for Rare Diseases, University Medical Center Hamburg-Eppendorf, Hamburg, Germany; 8 Siemens Healthcare, Los Angeles, California, United States; Istituto Di Ricerche Farmacologiche Mario Negri, ITALY

## Abstract

**Background and Purpose:**

Conventional magnetic resonance imaging (MRI) of patients with hemolytic uremic syndrome (HUS) and neurological symptoms performed during an epidemic outbreak of Escherichia coli O104:H4 in Northern Europe has previously shown pathological changes in only approximately 50% of patients. In contrast, susceptibility-weighted imaging (SWI) revealed a loss of venous contrast in a large number of patients. We hypothesized that this observation may be due to an increase in cerebral blood flow (CBF) and aimed to identify a plausible cause.

**Materials and Methods:**

Baseline 1.5T MRI scans of 36 patients (female, 26; male, 10; mean age, 38.2±19.3 years) were evaluated. Venous contrast was rated on standard SWI minimum intensity projections. A prototype four-dimensional (time resolved) magnetic resonance angiography (4D MRA) assessed cerebral hemodynamics by global time-to-peak (TTP), as a surrogate marker for CBF. Clinical parameters studied were hemoglobin, hematocrit, creatinine, urea levels, blood pressure, heart rate, and end-tidal CO_2_.

**Results:**

SWI venous contrast was abnormally low in 33 of 36 patients. TTP ranged from 3.7 to 10.2 frames (mean, 7.9 ± 1.4). Hemoglobin at the time of MRI (n = 35) was decreased in all patients (range, 5.0 to 12.6 g/dL; mean, 8.2 ± 1.4); hematocrit (n = 33) was abnormally low in all but a single patient (range, 14.3 to 37.2%; mean, 23.7 ± 4.2). Creatinine was abnormally high in 30 of 36 patients (83%) (range, 0.8 to 9.7; mean, 3.7 ± 2.2). SWI venous contrast correlated significantly with hemoglobin (r = 0.52, P = 0.0015), hematocrit (r = 0.65, P < 0.001), and TTP (r = 0.35, P = 0.036). No correlation of SWI with blood pressure, heart rate, end-tidal CO_2_, creatinine, and urea level was observed. Findings suggest that the loss of venous contrast is related to an increase in CBF secondary to severe anemia related to HUS. SWI contrast of patients with pathological conventional MRI findings was significantly lower compared to patients with normal MRI (mean SWI score, 1.41 and 2.05, respectively; P = 0.04). In patients with abnormal conventional MRI, mean TTP (7.45), mean hemoglobin (7.65), and mean hematocrit (22.0) were lower compared to patients with normal conventional MRI scans (mean TTP = 8.28, mean hemoglobin = 8.63, mean hematocrit = 25.23).

**Conclusion:**

In contrast to conventional MRI, almost all patients showed pathological changes in cerebral hemodynamics assessed by SWI and 4D MRA. Loss of venous contrast on SWI is most likely the result of an increase in CBF and may be related to the acute onset of anemia. Future studies will be needed to assess a possible therapeutic effect of blood transfusions in patients with HUS and neurological symptoms.

## Introduction

Hemolytic uremic syndrome (HUS), a severe complication of an infection with enterohemorrhagic Escherichia coli (EHEC), is typically observed in children. In contrast, young adults were mainly affected during an outbreak in 2011 in Northern Europe caused by a highly virulent and resistant strain of Escherichia coli O104:H4 [1–7]. Nearly half of the patients presented with neurological symptoms including headaches, delirium, cognitive dysfunction, aphasia, and epileptic seizures [8]. We have previously reported the most common magnetic resonance imaging (MRI) findings observed in patients infected with Escherichia coli O104:H4 [9]. These included bilateral symmetric signal abnormalities of thalamus, pons, central white matter, and splenium of corpus callosum on T2-weighted and fluid-attenuated inversion recovery images. Some lesions were characterized by restricted water diffusion suggestive of cytotoxic edema [9]. At baseline, approximately 50% of patients with neurological symptoms had abnormalities by conventional MRI. Findings were reversible in 81% of cases [9] and only three patients of a multi-center cohort suffered from relevant neurological deficits (cortical blindness, aphasia, cognitive deficits) eight months after clinical presentation [8].

**Fig 1:**
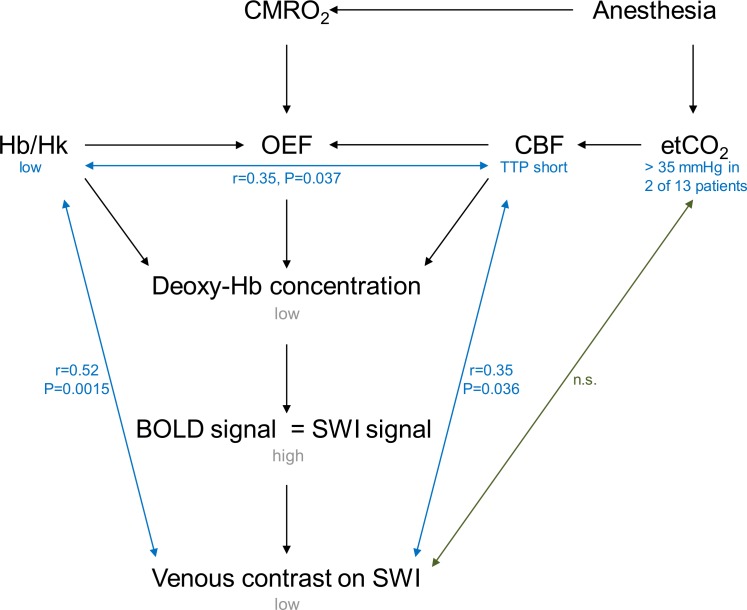
Schematic depicting factors which may influence the venous contrast on SWI. Note: CMRO_2_ = cerebral metabolic rate of oxygen, Hb = hemoglobin, Hk = hematocrit, OEF = oxygen extraction fraction, CBF = cerebral blood flow, TTP = time-to-peak, etCO_2_ = end-tidal CO_2_, BOLD = blood oxygen level dependent, SWI = susceptibility-weighted imaging, n.s. = not significant.

In addition to standard anatomical sequences, susceptibility-weighted imaging (SWI) and four-dimensional (time resolved) magnetic resonance angiography (4D MRA) were performed at our center. The rationale to include SWI was the detection of hemorrhagic brain lesions because anecdotal autopsy data suggests that parenchymal hemorrhage (i.e., petechial hemorrhage, hemorrhagic infarcts) and subdural hematomas are a common finding in HUS [10]. In addition to its high sensitivity to hemorrhage and calcification, SWI is a blood oxygen level dependent (BOLD) technique, which allows an assessment of the venous oxygenation level. In our cohort, subdural hematomas, large parenchymal lesions or hemorrhagic infarcts were not present and only two patients showed small petechial hemorrhages [9]. However, SWI maps revealed a loss of venous contrast in a large number of neurologically impaired patients. Because many of these patients with HUS and severe neurological symptoms had normal MRI findings, we hypothesized that the low venous contrast (i.e., high venous oxygenation level) may be related to an impaired cerebral oxygen metabolism. However, many variables may influence the venous contrast on SWI. First, an increase in cerebral blood flow (CBF) causes a decreased venous contrast on SWI (Fig 1). The higher inflow of oxygenated blood to the brain results in a lower oxygen extraction fraction (OEF) and consequently lower deoxyhemoglobin concentrations. As a result, the blood oxygen level dependent signal increases. An increase in CBF may also be caused by low hematocrit levels [11] or etCO_2_ levels above 30–35 mmHg during general anesthesia with propofol in free breathing patients [12]. Apart from this, narcotic agents may directly influence the cerebral metabolic rate of oxygen (CMRO_2_) but also increase (e.g., barbiturates) or decrease (e.g., halothane, isoflurane) CBF [13]. Therefore, 4D MRA was included in the imaging protocol to assess cerebral hemodynamics and at the same time avoid the administration of a gadolinium-based contrast agent in our patients with severe impairment of renal function.

The purpose of our study was to identify a possible cause for the loss of venous contrast on SWI in neurologically symptomatic patients with HUS due to an infection with Escherichia coli O104:H4. We hypothesized that the loss of venous contrast on SWI is caused by an increased CBF.

## Materials and Methods

### Patients

During a local outbreak of EHEC O104:H4 in Germany between May and August 2011, patients with HUS and neurological symptoms received MRI scans of the brain to rule out infarction and bleeding. The MRI protocol was designed prospectively and was used for all patients studied. The data collection followed the guidelines of the Hamburg Board of Physicians in compliance with the Declaration of Helsinki and was approved by its Ethics Committee. All participants provided their written informed consent for participation in the study and the Ethics Committee of the Hamburg Board of Physicians approved the consent procedure.

The diagnosis of EHEC was established based on the following criteria: bloody diarrhea, vomiting or bowel cramps, and/or microbiological identification of Shiga-toxin-producing Escherichia coli. HUS was defined as thrombocytopenia (platelet count < 150 000/cm^3^), hemolytic anemia, and acute renal dysfunction (increase in serum creatinine level > 50%).

This study focused on advanced MRI sequences, specifically SWI and 4D MRA, in patients with HUS and neurological symptoms. We have previously reported conventional MRI in a multi-center cohort study which included some of our patients [9]. Neurological symptoms were defined in the same fashion as reported previously [8]. All patients with neurological symptom were scanned by MRI at one point during their stay in our hospital. Due to the acute situation during the outbreak, the time of MR with respect to onset of clinical symptoms varied (Table 1). MRI was unavailable for neurologically unobtrusive patients suffering from an infection with EHEC O104:H4 due to capacity limitations in view of the very large number of infected patients at our site. Of 51 patients who had an MRI performed at our hospital, three patients (ID 15, 20, 36) were excluded from the study because an infection with EHEC O104:H4 could not be confirmed later. SWI was not acquired in five patients. An additional 4 patients did not have 4D MRI performed and in another three patients 4D MRA could not be analyzed quantitatively due to motion artifacts. Consequently, 36 patients (female, 26; male, 10; mean age 38.2±19.3 years) were included in the final analysis (Table 1). Of these 36 patients, 22 patients had an EEG performed.

**Table 1:** Patient demographics, MRI findings and results of SWI and 4D MRA.

ID	Sex	Age[years]	Reason for exclusion from data analysis	First neurological symptom to MRI [days]	Signal changes on MRI (0–6)	etCO2 [mmHg]	SWI venous contrast [a.u.] (see Table 2)	TTP[frames]
1*	m	55.2		8	7	34	1	9.00
2*	f	56.4		3	6	31	1	5.17
3*	f	67.2		9	6	31	1	3.73
4*	m	32.1		4	7	32	1	8.26
5*	f	56.2		5	1	28	1	8.22
6*	f	39.0		6	6	26	1	9.54
7*	f	21.3		0	1	30	4	8.68
8*	f	29.8		3	6	39	2	8.07
9*	m	75.6		7	0	31	3	10.18
10	m	12.2		2	6	n/a	1	5.39
11	f	10.6		15	0	n/a	3	6.60
12*	m	5.3		1	6	n/a	1	7.07
13	f	25.8		2	6	n/a	1	5.31
14*	f	20.5		2	6	40	1	6.65
16*	f	31.9		7	6	29	1	9.00
17	f	40.9		0	0	n/a	2	8.43
18	m	66.5		2	0	n/a	3	8.94
19*	f	36.5		5	6	18	2	9.36
21	f	22.4		18	0	n/a	2	7.92
22	f	41.7		3	6	n/a	2	5.86
27*	f	65.3		7	0	33	1	8.64
28	f	31.1		8	7	n/a	2	8.66
31	f	60.9		9	0	n/a	1	9.06
32	f	39.3		9	0	n/a	1	8.80
34*	f	5.4		5	0	n/a	1	7.09
37	m	44.2		8	0	n/a	4	8.87
38	m	70.0		0	0	n/a	4	9.64
39	f	40.7		8	0	n/a	2	7.60
41	m	28.5		13	0	n/a	1	7.83
42	m	25.3		8	0	n/a	1	7.56
43	m	26.1		9	0	n/a	1	7.73
45	f	29.8		17	0	n/a	1	8.78
46	m	53.9		9	0	n/a	2	8.42
47	f	27.1		14	0	n/a	2	8.22
48	m	66.0		5	0	n/a	3	8.81
50	f	13.4		22	0	n/a	2	6.76
Patients excluded (below)								
15	f	71.0	no EHEC					
20	m	67.2	no EHEC					
23	f	26.4	no 4D MRA	3	0	n/a	2	n/a
24	m	38.3	no 4D MRA	1	0	n/a	1	n/a
25	f	26.2	no SWI/4D MRA	0	n/a	n/a	n/a	n/a
26	m	63.0	no SWI/4D MRA	2	0	n/a	n/a	n/a
29	f	35.0	no SWI/4D MRA	19	7	n/a	n/a	n/a
30*	m	37.5	no 4D MRA	1	6	39	3	n/a
33	m	75.0	4D MRA artifacts	3	0	n/a	2	n/a
35	f	67.1	no 4D MRA	10	0	n/a	1	n/a
36	m	31.5	no EHEC					
40	f	80.0	4D MRA artifacts	n/a	7	n/a	2	n/a
44	f	11.8	4D MRA artifacts	n/a	0	n/a	1	n/a
49	f	14.3	no SWI/4D MRA	n/a	0	n/a	n/a	n/a
51	f	23.0	no SWI	n/a	0	n/a	n/a	7.03

Note

* = patients scanned under general anesthesia, m = male, f = female, etCO_2_ = end-tidal CO_2_, SWI = susceptibility weighted imaging, a.u. = arbitrary unit, TTP = time to peak, signal changes on MRI: 0 –none, 1 –thalamus, 2 –pons, 3 –semiovale, 4 –corpus callosum, 6 –combination, 7 –other.

SWI was added to the study protocol for the purpose of identifying possible cerebral microhemorrhages and 4D MRA was added to the study protocol to assess global cerebral hemodynamics. Unfortunately, perfusion arterial spin labeling MRI was not available on our scanner at the time of the local outbreak of EHEC O104:H4 and we refrained from administering a gadolinum-based contrast agent because of severe renal dysfunction in our patients.

### Anesthesia

MRI was performed under general anesthesia in 15 patients (Table 1, asterisk). General anesthesia was initiated on the intensive care unit in all cases and continued by continuous infusion of sulfentanil and propofol. During MRI, sedation was considered insufficient when increases in heart rate or blood pressure were observed, or the patient was fighting the respirator. In such cases, a bolus of 40 mg propofol was administered and the infusion rate was increased, starting with sulfentanil at an initial dose of 30 μg/h and propofol at 3mg/kg body weight per hour until a stable sedation was achieved. Patients were ventilated via endotracheal tubes with a tidal volume of 6–8 ml/kg body weight and the frequency was adjusted to reach an expiratory CO_2_ of 35 mm Hg or less. Hemodynamics were stabilized with continuous infusion of norepinephrine as needed to reach a mean arterial pressure of 70 mmHg.

### Clinical chemistry

Hemoglobin levels (normal range, 14–17 g/dL) at the time of MRI were available for 35 of 36 patients and hematocrit (normal range, 36–48%) was available for 34 of 36 patients. Both parameters were retrospectively collected from patient charts. Special care was taken to select the closest time point to the MRI scan (3–5 hours for the patients scanned under general anesthesia, max. 24 hours for all other patients).

In addition, blood pressure [mm Hg] and heart rate (HR) [bpm] were available for 16 patients. Thirteen of those patients were scanned under general anesthesia. Therefore, etCO_2_ [mm Hg] was also recorded during MRI.

### Imaging parameters

Since all patients presented with renal failure, a gadolinium-based contrast agent was not used. Traditional arterial spin labeling perfusion measurement techniques were not available on the specific scanner during the time of the outbreak. Therefore, an available alternative arterial spin labeling technique, a prototype 4D non-contrast enhanced MRA sequence [14], was added to the protocol. This method is optimized to assess cerebral hemodynamics.

All MRI scans were performed on a 1.5T scanner (Magnetom Avanto, Siemens Healthcare GmbH, Erlangen, Germany). The scanner was also equipped with capabilities to monitor patients and to perform general anesthesia. The imaging protocol consisted of standard anatomical sequences, as well as SWI and 4D MRA. SWI was performed using the following parameters: echo time/repetition time/flip angle = 40 ms/56 ms/20°, matrix 320×260, slice thickness 2 mm. 4D MRA was performed as follows: echo time/repetition time/flip angle = 1.8 ms/60 ms/25°, slice thickness 1.5 mm, matrix 176×176, 12 frames. The global time-to-peak (TTP) of the inflowing labeled spins was calculated (see below) to assess the global cerebral hemodynamics. TTP measures blood flow velocity and is inversely correlated with cerebral blood flow (CBF).

### Hemodynamic analysis of non-contrast enhanced 4D MRA image sequences

The non-contrast enhanced 4D MRA image sequence [14] was used as basis for an analysis of the macro-vascular blood flow properties.

For data analysis, each 4D MRA image series was first reduced to a 3D temporal maximum intensity projection by calculating the maximal intensity over time for each voxel. This temporal projection leads to an advanced representation of the cerebrovascular system since it does not depend on the bolus arrival time. This is especially beneficial for an automatic segmentation of the cerebrovascular network, which was performed using a multi-step segmentation framework described previously [15,16]. The resulting vessel segmentation was used as basis for the analysis of the signal intensity curves of the non-contrast enhanced 4D MRA image sequences. For this purpose, 250 signal intensity curves were randomly selected from each non-contrast enhanced 4D MRA image sequence, whereas only curves of voxels part of the cerebrovascular segmentation were considered for this purpose. These 250 curves were used for the generation of a mean reference curve using an adapted version of the reference-based linear curve fit approach [17]. In contrast to the original approach, an iterative method was used in this work to calculate an unbiased reference curve. Therefore, a mean linear transformation, including shifting and scaling of time dimension and signal magnitude dimension, was calculated by adapting all n-1 curves to each curve using curve fitting principles. A B-spline interpolation was used to enable an estimation of the signal intensity curves between the discrete sample points. After this, the mean transformation was calculated for each curve by averaging the inverted curve fit parameters. After transforming each curve using the corresponding calculated mean transformation, a B-Spline approximation was used to extract the final reference curve based on the point cloud consisting of the transformed discrete sample points. The reference curve exhibits a smooth shape, which allows the calculation of global TTP for statistical analysis. Finally, the extracted reference curve can also be used to estimate the TTP for each voxel included in the cerebrovascular segmentation by fitting the reference curve to each curve and transforming the TTP according to the transformation parameters. This allows generating 4D blood flow visualizations using the method described previously [18], which allows an intuitive and fast rating of the systemic blood flow situation.

### Data evaluation

**Fig 2:**
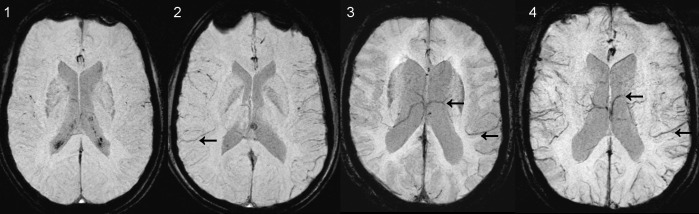
Rating scale for SWI venous contrast. 1 –no veins visualized, 2 –medium contrast of cortical veins (arrow), no deep veins, 3 –good contrast of cortical veins, medium contrast of deep veins (arrows), 4 –relatively preserved venous contrast (arrows).

The venous contrast on SWI was scored visually using the minimum intensity projection SWI and a modified scoring system described previously [12]. Venous contrast scores ranged from 1 to 4; 1 representing very low contrast (no veins visible) and 4 representing high contrast (good visibility of veins) (Table 2, Fig 2).

**Table 2:** Scoring of SWI venous contrast.

Score	Visibility of deep venous vasculature	Visibility of cortical veins
1	No	No
2	No	Medium
3	Medium	Good
4	Good	Good

### Statistics

SWI venous contrast of all patients was correlated using Pearson correlation coefficient (r) to TTP, hemoglobin, hematocrit, creatinine and urea. In a subset of patients based on availability of data, SWI venous contrast was correlated to etCO_2_, blood pressure and heart rate. Furthermore, TTP was correlated to hemoglobin and hematocrit.

Two-tailed Mann-Whitney U test was used for group comparison of patients scanned with and without anesthesia with respect to hemoglobin concentration, SWI venous contrast, and TTP. In addition, TTP was tested for a possible correlation with etCO_2_ in patients scanned under anesthesia.

Further, a group comparison was performed for patients with normal and abnormal MRI by conventional imaging with respect to hemoglobin concentration, SWI venous contrast, and TTP.

P values below 0.05, two-tailed, were considered statistically significant.

## Results

### Clinical findings

Patients initially presented with encephalopathy (17 patients), seizures (7 patients), aphasia (5 patients), headaches (3 patients), oculomotor dysfunction and myoclonus (2 patients each). EEG was abnormal in 18 of 22 patients with available EEG (S1 Table). We did not observe focal changes in this cohort and only one patient presented with epileptiform discharges. Most patients (12 of 18 patients with pathological MRI) showed general EEG changes.

### Hemoglobin, blood pressure, heart rate, creatinine, urea levels, and etCO_2_

Hemoglobin levels were decreased in all patients with available data at the time of MRI (n = 35), ranging from 5.0 to 12.6 g/dL (mean, 8.2±1.4). Also, hematocrit (n = 33) was abnormally low in all but a single patient (range, 14.3 to 37.2%; mean 23.7±4.2). Patient 21 did not have blood work performed on the day of MRI; therefore, hemoglobin and hematocrit on admission to hospital are given (S2 Table). Systolic and diastolic blood pressure of 16 anesthetized patients ranged from 98 to 194 mmHg (mean, 138±25), and from 47 to 89 mmHg (mean, 69±12), respectively. Mean heart rate ranged from 61 to 105 bpm (mean, 83±13). Creatinine was abnormally high in 30 of 36 patients (83%) ranging from 0.8 to 9.7 (mean, 3.7±2.2). Urea levels were increased in 28 of 36 patients (78%) ranging from 7.0 to 132.0 (mean, 49.3±32.6). (S2 Table). Based on 13 anesthetized patients, etCO_2_ during the MRI scans ranged from 18 to 40 mmHg (mean, 30.9±5.5) with only two patients showing increased etCO_2_ levels above 35 mmHg (Table 1).

### MR imaging

Conventional MRI was normal in 19 of 36 patients (53%) with neurological symptoms. Results of conventional MRI findings are given in Table 1 (for a more detailed evaluation see [9]).

SWI did not reveal hemorrhagic lesions in any of our patients. However, venous contrast on SWI was low in 33/36 patients (91.7%) with 19 patients (52.8%) showing a complete loss of venous contrast (Table 1). Cortical veins were visible in 10 patients only (27.8%). Using 4D MRA, global TTP ranged from 3.7 to 10.2 frames (mean, 7.9±1.4) with a temporal resolution of 60 ms per frame (Table 1).

### Statistics

**Fig 3:**
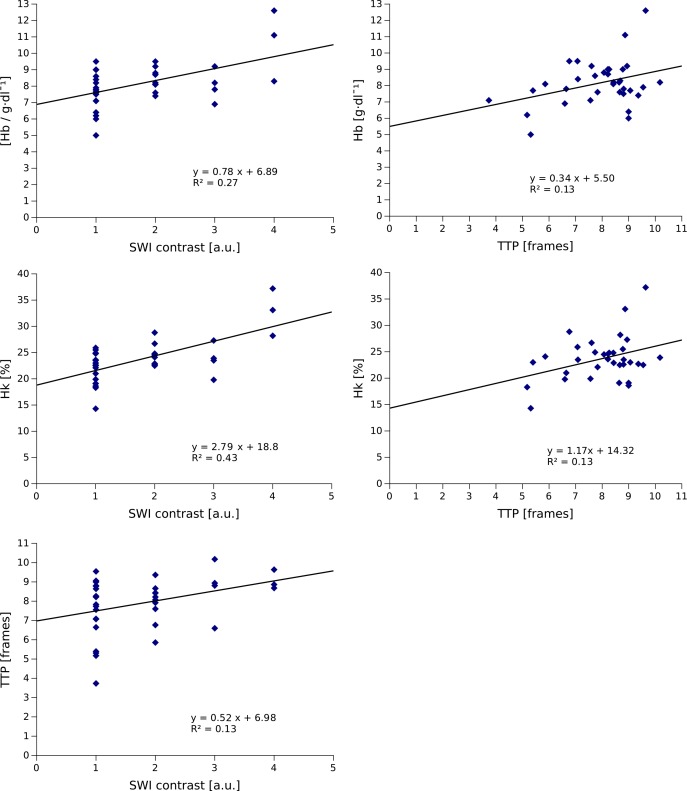
Results of statistical analysis. SWI venous contrast correlated significantly with hemoglobin, hematocrit, and TTP. TTP correlated significantly with hemoglobin and hematocrit.

**Fig 4:**
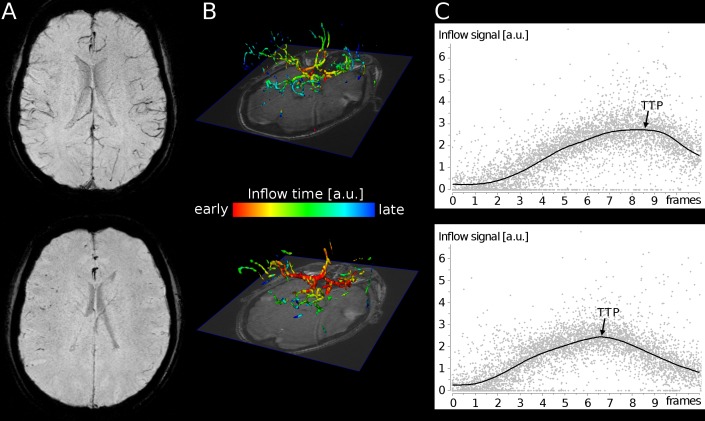
Patient examples. (A) SWI minimum intensity projection image, (B) 4D visualization of local inflow time-to-peak (TTP) and (C) global inflow curve depicting the global TTP. The first patient (top row) with good SWI venous contrast is a 21-year-old female (P07; SWI score, 4; etCO_*2*_ = 30 mmHg) showing shorter TTP by 4D MRA. Patient 2 (bottom row), a 20-year-old female (P14; SWI score, 1; etCO_*2*_ = 40 mmHg), shows decreased venous contrast on SWI and prolonged TTP. *Note*: *a*.*u*. *= arbitrary units*.

We identified a significant correlation of SWI venous contrast score (1–4) with hemoglobin (r = 0.52, P = 0.0015), and TTP (r = 0.35, P = 0.036) (Fig 3). Correlations of SWI venous contrast with etCO_2_, systolic and diastolic blood pressure, heart rate, creatinine, and urea levels were not statistically significant. Patients with good venous contrast on SWI show longer TTP values (Fig 4, top row), while patients with complete loss of venous contrast revealed shorter TTP values (Fig 4, bottom row). In addition, TTP correlated significantly with hemoglobin levels (r = 0.35, P = 0.037) and hematocrit (r = 0.36, P = 0.038) (Fig 3). Correlations of TTP with etCO_2_ were not statistically significant.

Mean TTP of patients scanned with anesthesia was 8.04 compared to 7.80 in patients without anesthesia. Similarly, hemoglobin of anesthetized patients was lower with a mean TTP of 7.72 compared to 8.41. However, there was no statistically significant difference between both groups for TTP, SWI venous contrast, and hemoglobin levels.

SWI contrast of patients with pathological conventional MRI findings was significantly lower compared to patients with normal MRI (mean SWI score, 1.41 and 2.05, respectively; P = 0.04). In patients with abnormal conventional MRI, mean TTP (7.45), mean hemoglobin (7.65), and mean hematocrit (22.0) were lower compared to patients with normal conventional MRI scans (mean TTP = 8.28, mean hemoglobin = 8.63, mean hematocrit = 25.23). This was statistically significant for hemoglobin (P = 0.03) and hematocrit (P = 0.02), but not for TTP (P = 0.09).

## Discussion

The evaluation of advanced MRI techniques in patients with HUS and neurological symptoms due to an infection with Escherichia coli O104:H4 showed low SWI venous contrast in nearly all patients (91.7%) Conversely, conventional MRI was abnormal in only 53% of patients. At the time of MRI, hemoglobin levels were decreased in 35 patients and hematocrit was low in 33 of 34 patients due to hemolytic anemia. Venous contrast on SWI correlated significantly with hemoglobin, hematocrit and TTP. Similarly, TTP showed a significant correlation to hemoglobin and hematocrit. In addition, a comparison of patients with and without brain lesions on conventional MRI resulted in significantly different values for venous contrast on SWI, hemoglobin levels and hematocrit, but not for TTP. Generally, venous contrast on SWI, hemoglobin and hematocrit were lower in patients with pathological findings on conventional MRI compared to patients without brain lesions. It is important to note that venous contrast on SWI did not correlate with etCO_2_ in patients scanned under general anesthesia. Also, etCO_2_ did not correlate with TTP values in this subgroup.

Our data suggest that the presence of anemia in our patients was associated with a compensatory increase in CBF to achieve an increase in oxygen supply to the brain [19]. In situations when this mechanism is able to compensate for low hemoglobin levels in the blood, both, CMRO_2_ and OEF, should remain stable. Since hemoglobin supplied to the brain was low and an increased CBF likely results in a faster transportation of deoxyhemoglobin away from the brain, deoxyhemoglobin concentration is likely decreased. This scenario could explain the low venous contrast on SWI observed in our patient cohort. It also shows that the loss of venous contrast on SWI is not a specific marker of an OEF change, unless hemoglobin levels are normal. Fig 1 shows that hemoglobin levels and CBF both influence the OEF. Based on our data, it is difficult to establish the impact of each parameter because OEF and CMRO_2_ were not measured. However, it is known that compensatory mechanisms to maintain CMRO_2_ may be exhausted at very low hemoglobin levels. To some extent this is related to the fact that a reduction in blood transit time limits the oxygen extraction of the brain because the surface area of capillaries cannot be increased any more. This relationship is termed the Buxton–Frank diffusion-limited model of oxygen delivery [13]. We have tried to explore this further by comparing patients with normal and abnormal conventional MRI scans and found that venous contrast on SWI, hemoglobin levels and hematocrit were significantly lower in patients with abnormal conventional MRI. In contrast, TTP was not significantly different, although TTP was slightly lower in patients with abnormal MRI. It can therefore be hypothesized that in patients with pathological findings on conventional MRI, the increase in cerebral blood flow was not enough to compensate for the low oxygen delivery to the brain causing neurological symptoms. Further studies will be needed for confirmation.

Our conclusion that anemia triggered a compensatory CBF increase is supported by the finding that etCO_2_ did not correlate with TTP values, which makes it unlikely that the loss of venous contrast on SWI was solely related to hypercapnia in patients scanned under general anesthesia. Hypercapnia with etCO_2_ levels above 30 to 35 mmHg [20] may cause loss of venous contrast on SWI in free breathing patients under sedation with propofol via an increase in CBF [12]. In our patients, etCO_2_ levels were below 35 mmHg in all but two patients.

Also, we did not identify a significant difference between patients scanned with and without anesthesia for SWI venous contrast and TTP. Therefore, a loss of venous contrast solely due to the application of narcotics (e.g., barbiturates, halothane, isoflurane [13]) and a consequent reduction of CMRO_2_ is unlikely. EEG changes can be the result of decreased brain activity, as previously reported in an experimental setting of anemia in rabbits [21]. The authors of the article measured increased CBF and oxygen extraction with increasing hemodilution/anemia, which supports the conclusion that the CBF increase observed in our patients is related to the underlying anemia. The authors further discuss that the increase in CBF only partially compensates the low arterial oxygen content because they observed a decrease in CMRO_2_ and abnormal findings in EEG at very high levels of hemodilution. Unfortunately, based on our data, we cannot conclude that EEG abnormalities or decreased levels of consciousness in our patients are a direct result of anemia and HUS because CMRO_2_ was not directly measured. However, a study of patients with subarachnoid hemorrhage and anemia who were treated with red blood cell transfusions observed improved oxygen delivery to the brain without significantly decreasing CBF [22]. Many of our patients did not have any co-morbidity, especially cardiovascular diseases or risk profile. Consequently, a restrictive transfusion regime was applied, and packed red blood cells were only given when physiological transfusion triggers were observed. Our data are not sufficient enough to conclude that neurological symptoms related to HUS may be considered as transfusion trigger in patients with severe anemia. This would be an interesting hypothesis for future studies. However, it has to be considered that a direct toxic effect of Shiga-toxin and a toxicity due to uremia may be present as well. More recently, disease severity and neurological involvement in patients with HUS due to an infection with EHEC has been linked to pathological levels of several cytokines, such as soluble TNF receptor 1, tissue inhibitor of metalloproteinase-1, angiopoietin 1 and 2 [23–25]. More specifically, angiopoietin 2 was reported to be significantly increased in patients with encephalopathy related to HUS but not in patients without encephalopathy [26]. Angiopoietin 2 is able to induce blood brain barrier breakdown. One study reported a correlation between levels of serum tau protein and MRI findings [27], suggesting that serum tau protein can assess disease severity in this patient cohort. Further studies will be needed to clarify the influence of cytokines, hemoglobin and hematocrit on encephalopathy and MRI findings in patients with HUS.

Also, it needs to be asserted that the finding of loss of venous contrast on SWI is not specific to an infection with enterohemorrhagic Escherichia coli O104:H4 but has also been described in patients with sickle cell disease [28] and multiple sclerosis [29].

Some limitations of the study need to be addressed. Although the study imaging protocols were designed in a prospective fashion, inclusion of patients into the study was related to clinical demands and availably of the scanner during the outbreak. This did not allow for scanning of HUS patients without neurological symptoms. Unfortunately, perfusion sensitive arterial spin labeling and quantitative susceptibility mapping MRI methods were not available at our hospital at the time of data collection. However, both methods are necessary for a quantitative MRI based calculation of CMRO_2_ [30–32].

## Conclusion

In contrast to conventional MRI which was abnormal in only about half of the patients with HUS and neurological symptoms, almost all patients showed changes in cerebral hemodynamics assessed by SWI and 4D MRA. Loss of venous contrast on SWI is most likely the result of an increase in CBF due to acute onset of anemia. The fact that hemoglobin was lower in patients with brain lesions compared to patients with normal conventional MRI suggests a relationship of clinical findings to the severity of anemia. Future studies will be needed to assess a possible therapeutic effect of blood transfusions in patients with HUS and neurological symptoms.

## Supporting Information

S1 Table Neurological characterization of patients included and excluded from the study. Note: n/a = not available; Initial neurological symptom: 0 –none, 1 –seizure, 2 –encephalopathy, 3 –aphasia, 4 –headaches, 5 –oculomotor symptoms, 6 –myoclonus; EEG changes: 0 –none, 1 –general changes, 2 –epileptiform discharges, 3 –focal changes, 4 –combination of changes; Encephalopathy, aphasia, paresis, cranial nerve deficits and apraxia: 0 –none, 1 –discrete, 2 –severe; Headache, seizures, myoclonus, and other symptoms: 0 –abscent, 1 –present.
(XLS)

S2 Table Clinical parameters including reference values. Note: f = female, m = male, n/a = not available, * = parameters obtained at the time of MRI were not available; values at presentation to the hospital are given (not included in the statistical analysis).
(XLS)

## Abbreviations

4D MRA: Four-dimensional (time resolved) magnetic resonance angiography
CBF: Cerebral blood flow
CMRO_2_: Cerebral metabolic rate of oxygen
EHEC: Enterohemorrhagic Escherichia coli
etCO_2_: End-tidal CO_2_
HUS: Hemolytic uremic syndrome
MRI: Magnetic resonance imaging
OEF: Oxygen extraction fraction
SWI: Susceptibility-weighted imaging
TTP: Time-to-peak
