# Correction: Cerebral Hemodynamics in Patients with Hemolytic Uremic Syndrome Assessed by Susceptibility Weighted Imaging and Four-Dimensional Non-Contrast MR Angiography

**DOI:** 10.1371/journal.pone.0172450

**Published:** 2017-02-14

**Authors:** Ulrike Löbel, Nils Daniel Forkert, Peter Schmitt, Thorsten Dohrmann, Maria Schroeder, Tim Magnus, Stefan Kluge, Christina Weiler-Normann, Xiaoming Bi, Jens Fiehler, Jan Sedlacik

The fourth author’s first name is spelled incorrectly. The correct name is: Thorsten Dohrmann.
